# Assessment of knowledge and skills in information literacy instruction for rehabilitation sciences students: a scoping review

**DOI:** 10.5195/jmla.2018.227

**Published:** 2018-01-02

**Authors:** Jill T. Boruff, Pamela Harrison

## Abstract

**Objective:**

This scoping review investigates how knowledge and skills are assessed in the information literacy (IL) instruction for students in physical therapy, occupational therapy, or speech-language pathology, regardless of whether the instruction was given by a librarian. The objectives were to discover what assessment measures were used, determine whether these assessment methods were tested for reliability and validity, and provide librarians with guidance on assessment methods to use in their instruction in evidence-based practice contexts.

**Methods:**

A scoping review methodology was used. A systematic search strategy was run in Ovid MEDLINE and adapted for CINAHL; EMBASE; Education Resources Information Center (ERIC) (EBSCO); Library and Information Science Abstracts (LISA); Library, Information Science & Technology Abstracts (LISTA); and Proquest Theses and Dissertations from 1990 to January 16, 2017. Forty articles were included for data extraction.

**Results:**

Three major themes emerged: types of measures used, type and context of librarian involvement, and skills and outcomes described. Thirty-four measures of attitude and thirty-seven measures of performance were identified. Course products were the most commonly used type of performance measure. Librarians were involved in almost half the studies, most frequently as instructor, but also as author or assessor. Information literacy skills such as question formulation and database searching were described in studies that did not involve a librarian.

**Conclusion:**

Librarians involved in instructional assessment can use rubrics such as the Valid Assessment of Learning in Undergraduate Education (VALUE) when grading assignments to improve the measurement of knowledge and skills in course-integrated IL instruction. The Adapted Fresno Test could be modified to better suit the real-life application of IL knowledge and skills.

## INTRODUCTION

Since the term evidence-based medicine was first coined in the mid-1990s [1], the integration of evidence-based practice (EBP) in health professional education has continued to grow. While EBP has its roots in medicine, it is now established in the required competencies of allied health professions such as physical therapy, occupational therapy, and speech-language pathology [2–4]. Along with this growth has come the establishment of information literacy (IL) instruction to support EBP [5–7].

The EBP cycle has been well defined in the literature: instructors in rehabilitation sciences tend to use a five-step model: (i) formulating the clinical question, (ii) searching the evidence, (iii) appraising the evidence, (iv) incorporating evidence into decision making, and (v) evaluating the process [1, 8–10]. Teaching and assessment in EBP education is categorized into knowledge, skills, attitudes, and behaviors [11–13] and can be applied to IL instruction. Knowledge in the context of EBP is “an awareness of the sources of evidence and an understanding of the evidence itself” [13]. Skills include the application of this knowledge [12]. An IL example would be the knowledge of how a database functions and the skill of how to conduct searches in this database. Attitudes refer to the recognition of the need to use certain knowledge or skills while working on an academic project or in clinical practice [11]. This differs slightly from attitude in the context of measures of attitude, which are self-reported perceived learning [14]. An EBP behavior is when someone “applies the knowledge and skills to solve [an] issue in practice” [11]. These categories help instructors align their teaching and assessment methods with learning outcomes.

IL knowledge and skills are necessary at every step of the EBP cycle. The Association of College and Research Libraries *Information Literacy Competency Standards* of “Determine, Access, Evaluate, Apply, and Ethics” correlate with the five EBP steps of “Formulate, Search, Appraise, Incorporate, Evaluate” [5] and provide a structure for teaching the IL knowledge and skills needed for EBP [6, 7]. Librarians are experts in such knowledge and skills as question formulation (patient-intervention-comparison-outcome [PICO] format and other research question formats), database searching, resource selection, article selection, and article appraisal (critical appraisal), and can be the model for future clinicians who will use such knowledge and skills in practice [15].

As recommended by good IL practice, librarians teaching in EBP contexts “routinely state specific instructional goals, explain rationales for teaching methods, identify ways they expect students to be impacted by IL instruction, and detail the effect of library instruction…on student success” [16]. This last point, examining the effect of instruction on student success, can be the most difficult to implement, though good assessment is important to help instructors understand if students are learning what the instructor intends them to learn [17].

Studies that looked at how librarians included assessment in their practice revealed that the assessment methods were primarily indirect assessments or assessment of attitude, which was defined as “self-reporting of perceived skills, learning, behaviors, or attitudes” [14]. Warner found in her review of the library assessment literature that there were “serious limitations to the instruments most typically used, primarily that they have failed to adequately assess student learning” [18]. In interviewing eighteen librarians, Cull observed that “most librarians…did not objectively assess student learning at all but only solicited student reactions to instructional content, methods, and instructor effectiveness, typically using brief end-of-class student evaluation forms” [19]. Two other studies also noted that self-reported attitude assessments were the most frequently cited form of evaluation in library instruction [14, 20].

While assessments of attitude are important for understanding how students feel about their own learning and about the instructor’s teaching, students are not good evaluators of their own skill levels [21–23]. It is important, therefore, to assess their learning with measures of performance that document knowledge, skills, or behaviors based on actual student work [14]. Another aspect to consider is whether an assessment measure has been tested for reliability and validity. Validated assessment measures increase the accuracy and the reliability of results, facilitate comparison across different studies [24], and allow a richer understanding of the effectiveness of the instruction.

The importance of well-developed assessment measures is clear, but applying this knowledge can be a challenge, since librarians and other EBP instructors may not know which assessment measures would best align with their learning outcomes and instructional methods. In an effort to assist instructors with this choice, literature reviews in library and information science and health sciences education have summarized the assessment measures being used in IL and EBP learning contexts, but not all educational contexts and measure types have been well addressed. These reviews leave major gaps in the literature.

The authors found two gaps in the library and information science literature. First, all of the reviews conducted by librarians looked only at studies where librarians were involved in the design or delivery of instruction and assessment [14, 25–29]. Given the alignment of IL instruction objectives and EBP instruction objectives [5–7], it is valuable to consider studies where instruction and assessment of IL outcomes took place without explicit librarian involvement, as these studies might provide librarians with examples of assessment methods used in health sciences education and novel opportunities for assessment.

Second, none of the reviews that librarians conducted covered the assessment of rehabilitation sciences students. The earliest review was conducted in 2006, before the Adapted Fresno Test for occupational therapy was created and does not have much content from rehabilitation sciences [25]. Two more recent reviews provided excellent overviews of the assessments that librarians were conducting but were broader in subject coverage, were narrower in date coverage, and did not provide specific recommendations for assessment measures in EBP instruction [14, 26]. Three other recent reviews specifically focused on instruction in health sciences but still left a gap for assessment in rehabilitation sciences. One included teaching methods but not assessment measures [27]; the other two recommended the Fresno Test, but no or few studies outside of medicine were included [28, 29].

We found one major gap in the health sciences, in which none of the reviews focused on the performance assessment of students in physical therapy, occupational therapy, or speech-language pathology. The reviews in medicine focused on studies with medical students and clinicians [11, 12], and the reviews in rehabilitation sciences focused on practicing occupational and physical therapists [13, 30] or only found measures of attitude [31]. Three reviews found the Fresno Test [12] or the Adapted Fresno Test [13, 30] as the only validated assessments of performance, but they were used with medical students or rehabilitation sciences clinicians. Assessments of rehabilitation sciences clinicians or of medical students and clinicians are not sufficient. Rehabilitation sciences professionals approach practice differently than medical professionals, and “discipline-specific measures that address a profession’s own EBP needs and concerns may be warranted” [13, 30]. Students will not have the same level of EBP skills and knowledge as an expert clinician, and assessments of students should reflect this difference [15].

To address these gaps in the literature, this scoping review investigates how knowledge and skills are assessed in the IL instruction of physical therapy, occupational therapy, and speech-language pathology students, regardless of whether the instruction was designed and delivered by a librarian.

This project had three main objectives: (1) to determine what assessment methods were being used, (2) to determine whether these assessment methods had been tested for reliability and validity, and (3) to provide librarians with guidance on assessment methods that could be used in their own instruction. We chose to report on both measures of attitude as well as measures of performance, though our focus was on the latter.

## METHODS

We chose a scoping review rather than a systematic review methodology for this review, because we addressed a broad question, “aiming to summarize and disseminate research findings” as proposed by Arksey and O’Malley in their definition and outlined in the stages below [32].

### Stage 1: Identifying the research question

The original research question stated: How are knowledge and skills being assessed in the IL instruction of nursing and allied health students? After the first round of full-text screening, we narrowed the scope to include only physical therapy, occupational therapy, and speech-language pathology students, and we removed nursing from the research question due to reasons explained in stage 3 below.

### Stage 2: Identifying relevant studies

A systematic search strategy was constructed by one author and reviewed by the second author as recommended by the PRESS standard [33]. This strategy was then run in MEDLINE (Ovid) and adapted for CINAHL; EMBASE (Ovid); Education Resources Information Center (ERIC) (EBSCO); Library and Information Science Abstracts (LISA); Library, Information Science & Technology Abstracts (LISTA); and Proquest Theses and Dissertations from 1990 to January 16, 2015. Four thousand ninety-seven articles were found, with 2,747 articles remaining after duplicates were removed. No limits for language or publication type were applied at the searching stage. EndNote citation software was used for duplicate removal and screening. The MEDLINE search strategy can be found in the supplemental appendix.

### Stage 3: Selecting studies

The two authors each independently screened the titles and abstracts of all 2,747 articles, resulting in the exclusion of 2,143 articles. We then screened the remaining 604 full-text articles for eligibility. A third librarian was available to resolve any disagreements that were not resolved by discussion between the 2 authors; however, external consultation was not needed.

To be included in the review, the studies had to describe the assessment of IL skills in undergraduate or graduate physical therapy, occupational therapy, speech-language pathology, nursing, or general allied health academic degree programs. We defined IL skills as any instruction on question formulation, searching, database use, critical appraisal, or any library skills that pertained to these aspects of EBP. A librarian did not have to be involved in the instruction or assessment, except in studies that focused exclusively on critical appraisal, in which case librarian involvement was a requirement for inclusion, in order to keep the focus on areas of librarian expertise.

We excluded articles if they were not in English or in French. One hundred thirty-five studies were initially included for data extraction. Due to the large number of included studies, we conducted a further round of screening to put aside the articles that pertained to nursing or allied health disciplines other than physical therapy, occupational therapy, or speech-language pathology.

**Figure 1:**
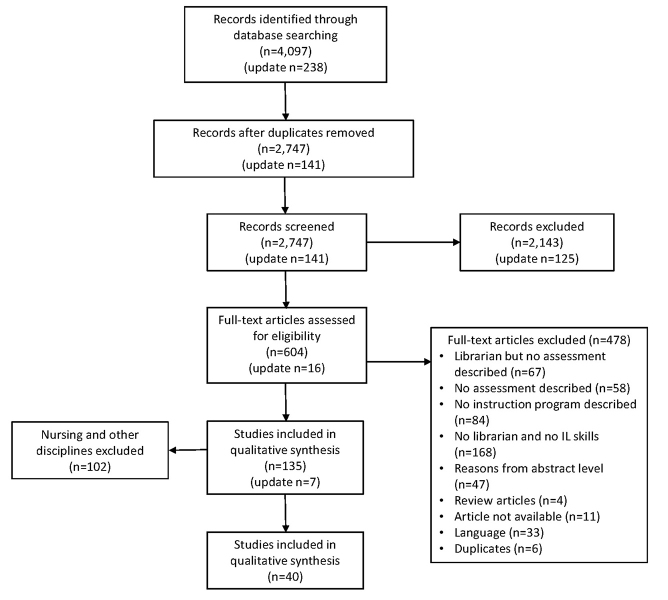
PRISMA flow diagram

This paper reports on the physical therapy, occupational therapy, and speech-language pathology portion of the data set. The final round of screening resulted in 33 articles for data extraction. For the update, the first author conducted a search from January 17, 2015, to January 16, 2017, and found 238 references, 141 after duplicates were removed. We screened these references using the new criteria, yielding 7 studies for inclusion. The PRISMA flow diagram (Figure 1) details the screening process [34].

### Stage 4: Charting the data

The two authors independently extracted the data from the forty included articles using an Excel spreadsheet with predefined fields for the following information in each study: country of study, degree level of learners, health profession of learners, librarian involvement (none or as instructor, assessor, author), measures of attitude used, measures of performance used, testing of measures for reliability or validity, instructional context of the IL instruction, knowledge and skills taught, and stated learning outcomes.

To determine whether the study assessed IL knowledge and skills, we had to explicitly define what IL knowledge and skills were important for EBP [15]. Using the study by Shaneyfelt et al. [12] as a model, and our own experience and research in EBP and IL to further refine, we used the following definitions while coding the articles:

Knowledge and/or skills in question formulation (PICO format and other research question formats); database searching (any mention of databases or the mechanics of searching); search quality (explicit content addressing appraisal of a search); search development (explicit content addressing how to develop a good search); resource selection (how to choose a database or another resource); article selection (content to select based on study design and relevance to topic); and article appraisal (critical appraisal)

To categorize the assessments found, we used the definitions in Schilling and Applegate:

Measures of performance are tests, course products, and portfolios; measures of attitude are self-report surveys, interviews, focus groups. [14]

We had hoped to report on whether the assessments were formative (in-process) measures or summative (at the end) and at what stage in the EBP cycle they took place, but the articles did not report enough information to make coding these data points possible.

We resolved any differences in data charting by discussion, with both authors returning to the original article to make sure that the data matched the article. No formal quality assessment was undertaken as the goal was to find all assessments being used, regardless of the quality of the study in which they were used.

### Stage 5: Collating, summarizing, and reporting the results

We used the data charted above to collate the data into descriptive statistics and summarize the qualitative themes that emerged. The qualitative themes arose from discussions between the two authors.

## RESULTS

Of the 40 included studies, the level of learners were undergraduate (n=15), graduate (n=19), or mixed undergraduate and graduate (n=6). Most of the studies were conducted in the United States (n=25) with other studies in Canada (n=4), Australia (n=5), continental Europe (n=3), Republic of Ireland (n=2), and the United Kingdom (n=1). The studies were evenly distributed across the included health professions: physical therapy (n=11), occupational therapy (n=11), mixed physical and occupational therapy programs (n=11), and speech-language pathology (n=7). Table 1 provides more information. Three major themes emerged as the authors discussed their findings: types of measures used, type and context of librarian involvement, and skills and outcomes described.

**Table 1:**
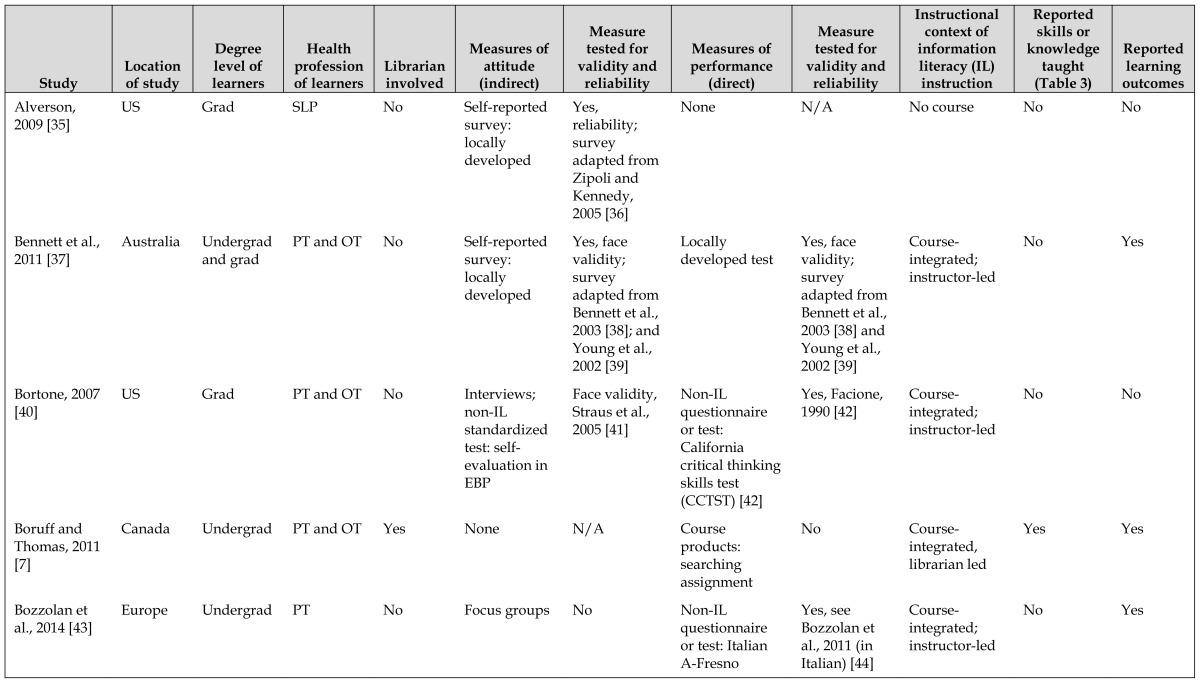

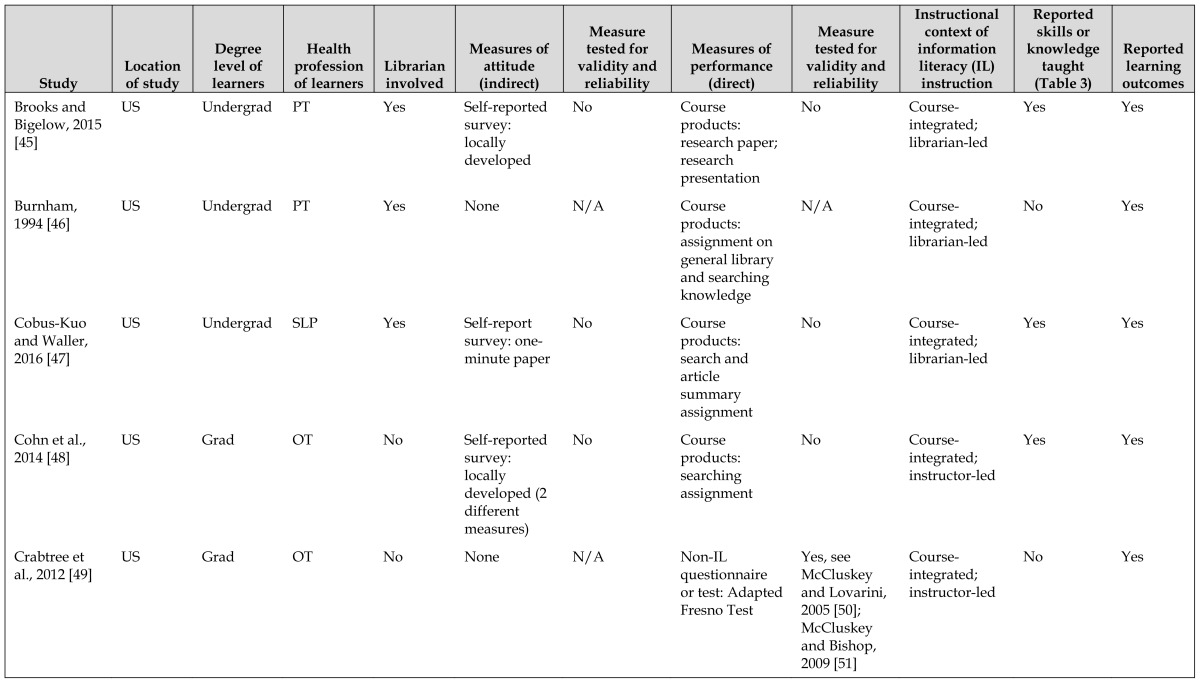

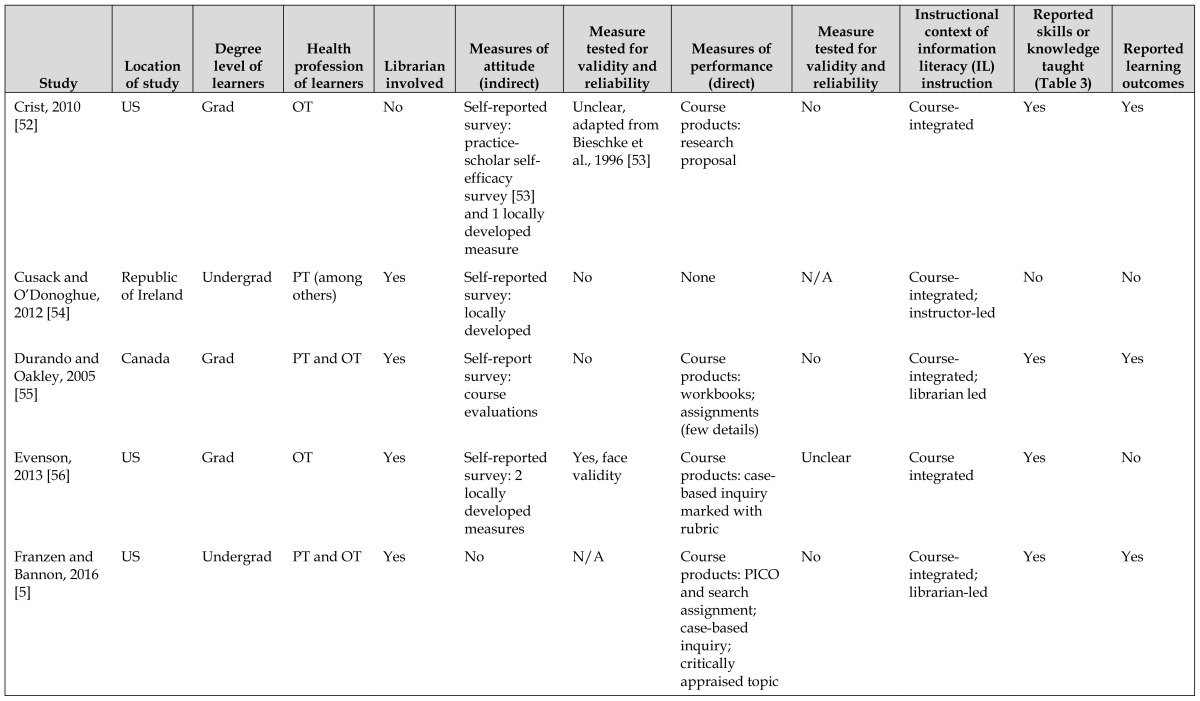

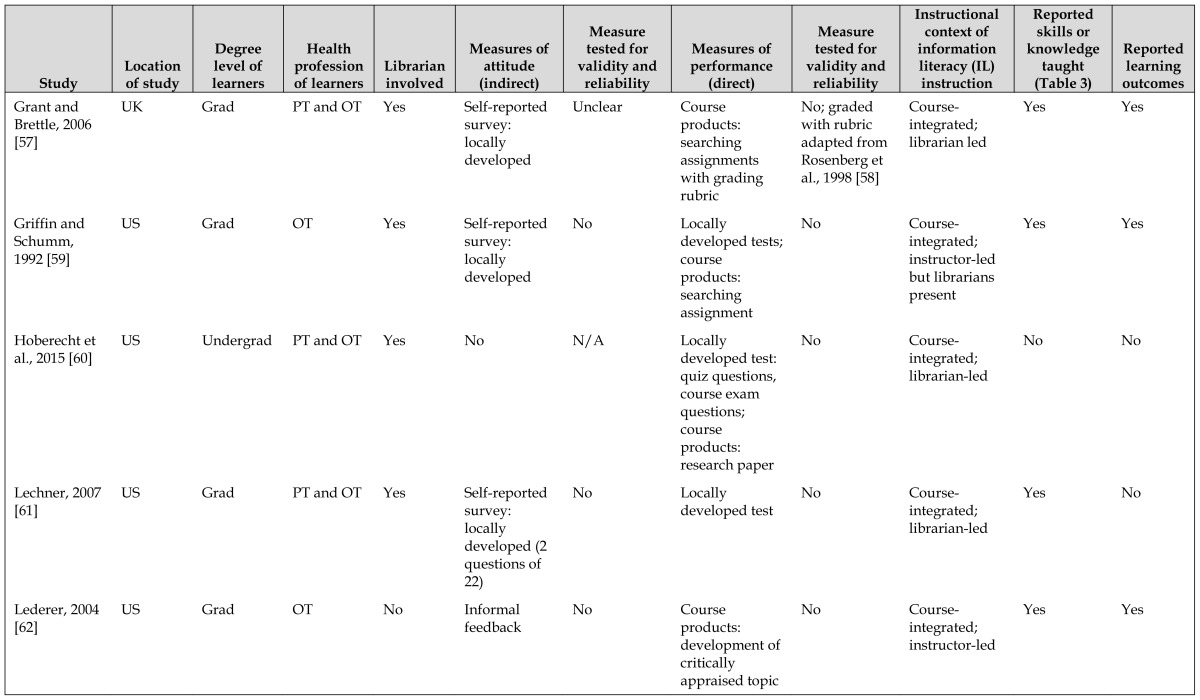

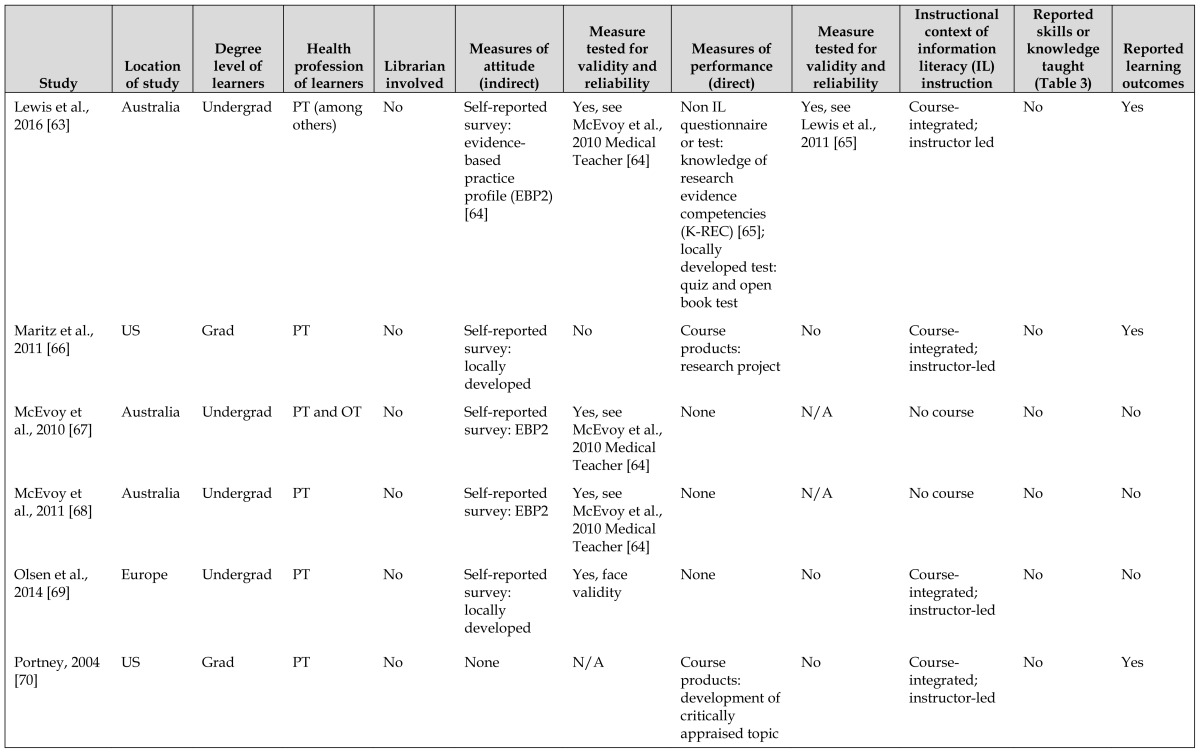

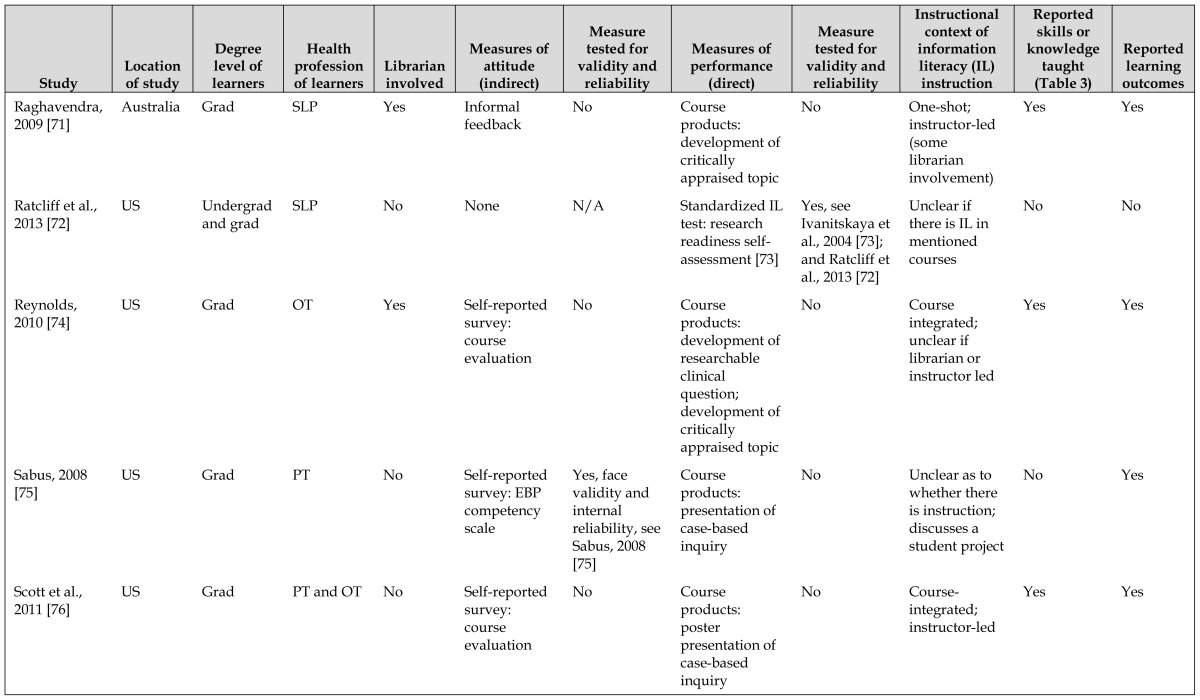

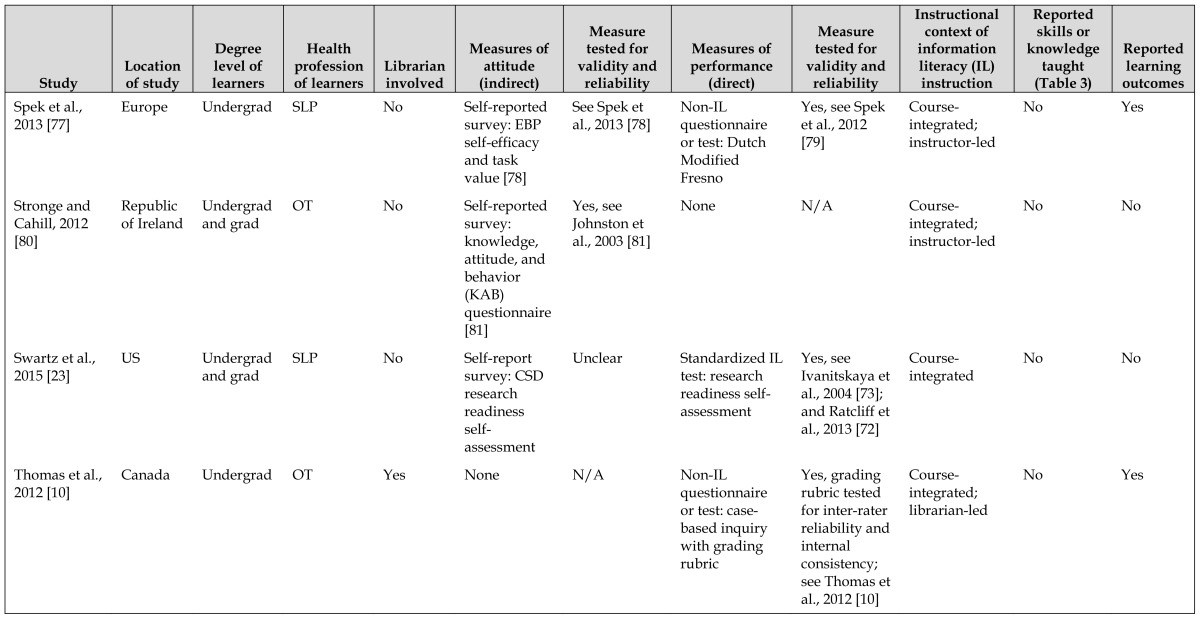
Included studies

Study	Location of study	Degree level of learners	Health profession of learners	Librarian involved	Measures of attitude (indirect)	Measure tested for validity and reliability	Measures of performance (direct)	Measure tested for validity and reliability	Instructional context of information literacy (IL) instruction	Reported skills or knowledge taught (Table 3)	Reported learning outcomes
Alverson, 2009 [35]	US	Grad	SLP	No	Self-reported survey: locally developed	Yes, reliability; survey adapted from Zipoli and Kennedy, 2005 [36]	None	N/A	No course	No	No
Bennett et al., 2011 [37]	Australia	Undergrad and grad	PT and OT	No	Self-reported survey: locally developed	Yes, face validity; survey adapted from Bennett et al., 2003 [38]; and Young et al., 2002 [39]	Locally developed test	Yes, face validity; survey adapted from Bennett et al., 2003 [38] and Young et al., 2002 [39]	Course-integrated; instructor-led	No	Yes
Bortone, 2007 [40]	US	Grad	PT and OT	No	Interviews; non-IL standardized test: self-evaluation in EBP	Face validity, Straus et al., 2005 [41]	Non-IL questionnaire or test: California critical thinking skills test (CCTST) [42]	Yes, Facione, 1990 [42]	Course-integrated; instructor-led	No	No
Boruff and Thomas, 2011 [7]	Canada	Undergrad	PT and OT	Yes	None	N/A	Course products: searching assignment	No	Course-integrated, librarian led	Yes	Yes
Bozzolan et al., 2014 [43]	Europe	Undergrad	PT	No	Focus groups	No	Non-IL questionnaire or test: Italian A-Fresno	Yes, see Bozzolan et al., 2011 (in Italian) [44]	Course-integrated; instructor-led	No	Yes
Brooks and Bigelow, 2015 [45]	US	Undergrad	PT	Yes	Self-reported survey: locally developed	No	Course products: research paper; research presentation	No	Course-integrated; librarian-led	Yes	Yes
Burnham, 1994 [46]	US	Undergrad	PT	Yes	None	N/A	Course products: assignment on general library and searching knowledge	N/A	Course-integrated; librarian-led	No	Yes
Cobus-Kuo and Waller, 2016 [47]	US	Undergrad	SLP	Yes	Self-report survey: one-minute paper	No	Course products: search and article summary assignment	No	Course-integrated; librarian-led	Yes	Yes
Cohn et al., 2014 [48]	US	Grad	OT	No	Self-reported survey: locally developed (2 different measures)	No	Course products: searching assignment	No	Course-integrated; instructor-led	Yes	Yes
Crabtree et al., 2012 [49]	US	Grad	OT	No	None	N/A	Non-IL questionnaire or test: Adapted Fresno Test	Yes, see McCluskey and Lovarini, 2005 [50]; McCluskey and Bishop, 2009 [51]	Course-integrated; instructor-led	No	Yes
Crist, 2010 [52]	US	Grad	OT	No	Self-reported survey: practice-scholar self-efficacy survey [53] and 1 locally developed measure	Unclear, adapted from Bieschke et al., 1996 [53]	Course products: research proposal	No	Course-integrated	Yes	Yes
Cusack and O’Donoghue, 2012 [54]	Republic of Ireland	Undergrad	PT (among others)	Yes	Self-reported survey: locally developed	No	None	N/A	Course-integrated; instructor-led	No	No
Durando and Oakley, 2005 [55]	Canada	Grad	PT and OT	Yes	Self-report survey: course evaluations	No	Course products: workbooks; assignments (few details)	No	Course-integrated; librarian led	Yes	Yes
Evenson, 2013 [56]	US	Grad	OT	Yes	Self-reported survey: 2 locally developed measures	Yes, face validity	Course products: case-based inquiry marked with rubric	Unclear	Course integrated	Yes	No
Franzen and Bannon, 2016 [5]	US	Undergrad	PT and OT	Yes	No	N/A	Course products: PICO and search assignment; case-based inquiry; critically appraised topic	No	Course-integrated; librarian-led	Yes	Yes
Grant and Brettle, 2006 [57]	UK	Grad	PT and OT	Yes	Self-reported survey: locally developed	Unclear	Course products: searching assignments with grading rubric	No; graded with rubric adapted from Rosenberg et al., 1998 [58]	Course-integrated; librarian led	Yes	Yes
Griffin and Schumm, 1992 [59]	US	Grad	OT	Yes	Self-reported survey: locally developed	No	Locally developed tests; course products: searching assignment	No	Course-integrated; instructor-led but librarians present	Yes	Yes
Hoberecht et al., 2015 [60]	US	Undergrad	PT and OT	Yes	No	N/A	Locally developed test: quiz questions, course exam questions; course products: research paper	No	Course-integrated; librarian-led	No	No
Lechner, 2007 [61]	US	Grad	PT and OT	Yes	Self-reported survey: locally developed (2 questions of 22)	No	Locally developed test	No	Course-integrated; librarian-led	Yes	No
Lederer, 2004 [62]	US	Grad	OT	No	Informal feedback	No	Course products: development of critically appraised topic	No	Course-integrated; instructor-led	Yes	Yes
Lewis et al., 2016 [63]	Australia	Undergrad	PT (among others)	No	Self-reported survey: evidence-based practice profile (EBP2) [64]	Yes, see McEvoy et al., 2010 Medical Teacher [64]	Non IL questionnaire or test: knowledge of research evidence competencies (K-REC) [65]; locally developed test: quiz and open book test	Yes, see Lewis et al., 2011 [65]	Course-integrated; instructor led	No	Yes
Maritz et al., 2011 [66]	US	Grad	PT	No	Self-reported survey: locally developed	No	Course products: research project	No	Course-integrated; instructor-led	No	Yes
McEvoy et al., 2010 [67]	Australia	Undergrad	PT and OT	No	Self-reported survey: EBP2	Yes, see McEvoy et al., 2010 Medical Teacher [64]	None	N/A	No course	No	No
McEvoy et al., 2011 [68]	Australia	Undergrad	PT	No	Self-reported survey: EBP2	Yes, see McEvoy et al., 2010 Medical Teacher [64]	None	N/A	No course	No	No
Olsen et al., 2014 [69]	Europe	Undergrad	PT	No	Self-reported survey: locally developed	Yes, face validity	None	No	Course-integrated; instructor-led	No	No
Portney, 2004 [70]	US	Grad	PT	No	None	N/A	Course products: development of critically appraised topic	No	Course-integrated; instructor-led	No	Yes
Raghavendra, 2009 [71]	Australia	Grad	SLP	Yes	Informal feedback	No	Course products: development of critically appraised topic	No	One-shot; instructor-led (some librarian involvement)	Yes	Yes
Ratcliff et al., 2013 [72]	US	Undergrad and grad	SLP	No	None	N/A	Standardized IL test: research readiness self-assessment [73]	Yes, see Ivanitskaya et al., 2004 [73]; and Ratcliff et al., 2013 [72]	Unclear if there is IL in mentioned courses	No	No
Reynolds, 2010 [74]	US	Grad	OT	Yes	Self-reported survey: course evaluation	No	Course products: development of researchable clinical question; development of critically appraised topic	No	Course integrated; unclear if librarian or instructor led	Yes	Yes
Sabus, 2008 [75]	US	Grad	PT	No	Self-reported survey: EBP competency scale	Yes, face validity and internal reliability, see Sabus, 2008 [75]	Course products: presentation of case-based inquiry	No	Unclear as to whether there is instruction; discusses a student project	No	Yes
Scott et al., 2011 [76]	US	Grad	PT and OT	No	Self-reported survey: course evaluation	No	Course products: poster presentation of case-based inquiry	No	Course-integrated; instructor-led	Yes	Yes
Spek et al., 2013 [77]	Europe	Undergrad	SLP	No	Self-reported survey: EBP self-efficacy and task value [78]	See Spek et al., 2013 [78]	Non-IL questionnaire or test: Dutch Modified Fresno	Yes, see Spek et al., 2012 [79]	Course-integrated; instructor-led	No	Yes
Stronge and Cahill, 2012 [80]	Republic of Ireland	Undergrad and grad	OT	No	Self-reported survey: knowledge, attitude, and behavior (KAB) questionnaire [81]	Yes, see Johnston et al., 2003 [81]	None	N/A	Course-integrated; instructor-led	No	No
Swartz et al., 2015 [23]	US	Undergrad and grad	SLP	No	Self-report survey: CSD research readiness self-assessment	Unclear	Standardized IL test: research readiness self-assessment	Yes, see Ivanitskaya et al., 2004 [73]; and Ratcliff et al., 2013 [72]	Course-integrated	No	No
Thomas et al., 2012 [10]	Canada	Undergrad	OT	Yes	None	N/A	Non-IL questionnaire or test: case-based inquiry with grading rubric	Yes, grading rubric tested for inter-rater reliability and internal consistency; see Thomas et al., 2012 [10]	Course-integrated; librarian-led	No	Yes
Turbow and Evener, 2016 [82]	US	Grad	PT (among others)	Yes	No	N/A	Course products: essay assignment	Yes, graded with adapted VALUE rubric; see Finley, 2011 [83]; and Rhodes and Finley, 2013 [84]	Course-integrated	No	No
Van Moorsel, 2005 [85]	US	Undergrad and grad	PT and OT	Yes	Self-reported survey: locally developed	No	Locally developed test; course products: research presentations	No	Course-integrated; librarian-led	Yes	Yes
Villeneuve and Maranda, 2005 [86]	Canada	Undergrad	OT	Yes	Focus group	No	Course products: case-based inquiry	No	2 × one-shot (1 OT session instructor-led, 1 IL session librarian-led)	Yes	Yes
Vogel, 2012 [87]	US	Grad	OT	Yes	Self-reported survey: locally developed	No	Course products: searching assignment	No	Course-integrated, librarian led	Yes	Yes
Wolter et al., 2011 [88]	US	Undergrad and grad	SLP	No	None	N/A	Course products: research article critique	No	Course-integrated	No	No

OT=occupational therapy.

PT= physical therapy.

SLP=speech-language pathology.

### Theme: Types of measures used

Thirty-four measures of attitude found in 30 studies were collated into the following categories: self-report surveys (n=25), focus groups (n=2), informal feedback (n=2), and interviews (n=1) [40]. Most of the self-report surveys were developed locally. Eight studies used one of the following published instruments: evidence-based practice profile (EBP2) [63, 67, 68]; knowledge, attitude, and behavior (KAB) questionnaire [80]; practice-scholar self-efficacy survey [52]; EBP competency scale [75]; self-evaluation in EBP [8]; and EBP self-efficacy and task value [77].

Thirty-seven measures of performance in 34 studies were collated into the following categories: course products (n=24); non-IL standardized tests, defined as tests developed for EBP contexts (n= 6, using 4 tests and 1 rubric); IL standardized tests, defined as tests developed for IL contexts (n=2, using the research readiness self-assessment [RRSA] [73]); and locally developed tests (n=5). The course products varied widely; for example, there were 7 searching assignments, where the search strategy was the main focus; 5 case-based inquiry [86] assignments, where students applied the 5 EBP steps to a patient scenario; 5 critically appraised topic assignments, where students summarized the evidence on a given topic; and 4 research project assignments. One study adapted the Valid Assessment of Learning in Undergraduate Education (VALUE) rubric to assess an EBP assignment [82].

The non-IL standardized tests were all originally tested for reliability and validity in separate published studies: the Adapted Fresno Test (for occupational therapy), the Dutch Modified Fresno Test (for speech-language pathology, in Dutch only), the knowledge of research evidence competencies (K-REC) test, and the California critical thinking skills test (CCTST). Table 2 provides more details on all of the published measures.

**Table 2:** Published assessment measures

Name of measure	Measure description	Studies where measure was tested for validity and reliability	Studies where measure was used
California critical thinking skills test (CCTST)	Created to measure critical thinking in university students taking critical thinking courses	Facione, 1990 [42]	Bortone, 2007 [40]
Fresno test	Designed to evaluate the effectiveness of a university evidence-based medicine curriculum for family practice residents	Ramos et al., 2003 [89]	No studies were found by this review, but this test served as the basis for other measures
Adapted Fresno Test (AFT)	Changed the original Fresno Test to include scenarios more relevant to occupational therapists and remove some statistical questions	McCluskey and Lovarini, 2005 [50]; McCluskey and Bishop, 2009 [51]	Crabtree et al., 2012 [49]
Italian A-Fresno	Translated the Adapted Fresno Test into Italian	Bozzolan et al., 2011 [44]	Bozzolan et al., 2014 [43]
Modified Fresno Test (MFT)	Changed the original Fresno Test to include scenarios relevant to physical therapy and to add 2 short answer questions	Tilson, 2010 [90]	No studies (other than the validation study) were found by this review
Dutch Modified Fresno	Translated the original Fresno Test into Dutch and changed it to include scenarios relevant to speech-language pathology	Spek et al., 2012 [79]	Spek, et al., 2013 [77]
Knowledge of research evidence competencies (K-REC)	Designed to test the cognitive skills of the first 3 steps of the EBP cycle in physical therapy	Lewis et al., 2011 [65]	Lewis et al., 2016 [63]
Research readiness self-assessment (RRSA)	Designed to assess the ability of university students as health information consumers to find and evaluate electronic health information; Ratcliff et al., 2013 [72]; and Swartz et al., 2015 [23], modified the language to emphasize speech-language pathology instead of general health topics	Ivanitskaya et al., 2004 [73]; Ratcliff et al., 2013 [72]	Ratcliff et al., 2013 [72]; Swartz et al., 2015 [23]
Valid Assessment of Learning in Undergraduate Education (VALUE) IL rubric	The IL rubric was 1 of 15 VALUE rubrics designed by the Association of American Colleges and Universities to assess learning outcomes based on student work; Turbow and Evener modified the rubric by replacing the word “information” with “evidence”	Rhodes, 2010 [84]	Turbow and Evener, 2016 [82]

### Theme: Type and context of librarian involvement

Librarian involvement was nearly equally divided, with almost half (n=19, 47.5%) of the studies involving a librarian. In these studies, the librarian was most frequently involved as an instructor (n=15), but the librarian was also involved as an author (n=14), as an assessor (n=12), and as a course designer (n=2). Though librarian involvement was not explicitly stated in the other half (n=21, 52.5%) of the studies, the students received instruction and/or assessment of searching and other IL skills. All but one of the included studies involving librarians used some measure of performance. The full breakdown of librarian involvement can be seen in Table 3.

**Table 3:** Librarian role in the study and assessment measures used

Study	Librarian as author	Librarian as instructor	Librarian as assessor	Librarian role unclear	Librarian as course designer	Used measure of attitude	Used measure of performance
Boruff and Thomas, 2011 [7]	X	X	X				X
Brooks and Bigelow, 2015 [45]	X	X	X		X	X	X
Burnham, 1994 [46]	X	X	X				X
Cobus-Kuo and Waller, 2016 [47]	X	X	X			X	X
Cusack and O’Donoghue, 2012 [54]					X	X	
Durando and Oakley, 2005 [55]	X	X	X			X	X
Evenson, 2013 [56]				X		X	X
Franzen and Bannon, 2016 [5]	X	X	X				X
Grant and Brettle, 2006 [57]	X	X	X			X	X
Griffin and Schumm, 1992 [59]	X	X				X	X
Hoberecht et al., 2015 [60]	X	X	X				X
Lechner, 2007 [61]	X	X	X			X	X
Raghavendra, 2009 [71]		X				X	X
Reynolds, 2010 [74]				X		X	X
Thomas et al., 2012 [10]		X					X
Turbow and Evener, 2016 [82]	X		X				X
Van Moorsel, 2005 [85]	X	X	X			X	X
Villeneuve and Maranda, 2005 [86]	X	X				X	X
Vogel, 2012 [87]	X	X	X			X	X

The majority of the studies (n=33, 82.5%) had IL knowledge and skills being taught in a course-integrated context by either a librarian or the instructor, though there were studies with one-shot instruction (n=2) and studies where the delivery context was unclear (n=2) or where no course was directly associated (n=3). While not all studies had clearly stated learning outcomes, 9 studies that did not describe librarian involvement included explicit statements regarding knowledge and skills often taught by a librarian, such as “effectively search research literature and judiciously select relevant evidence” [48] and “enable students to develop skills in formulating answerable clinical questions and finding and evaluating the research evidence” [70]. Of the 3 studies that used the Adapted Fresno Test or the Modified Dutch Fresno Test as the assessment for EBP knowledge and skills, both of which include a section on assessing the “finding the evidence” portion of the EBP cycle, none of the studies described the involvement of a librarian.

### Theme: Skills and outcomes described

Eighteen of the studies described the instructional content of the lectures, workshops, and course materials, and another 8 studies did not describe the content but had learning outcomes that described EBP knowledge and skills. For the purpose of summarizing the knowledge and skills taught, we merged the descriptions of instructional content and of learning outcomes, even though stating in an outcome that a course will teach certain concepts does not necessarily mean that the stated knowledge or skill has been taught. We have reported the specific knowledge and skills (as described in the methods) in Table 4. Most of these 26 studies (n=23, 88.5%) described database searching knowledge or skills, including 9 studies that did not involve a librarian. The second most common content topic of these 26 studies was question formulation (n=21, 80.8%), with librarians involved in 10 of the studies.

**Table 4:**
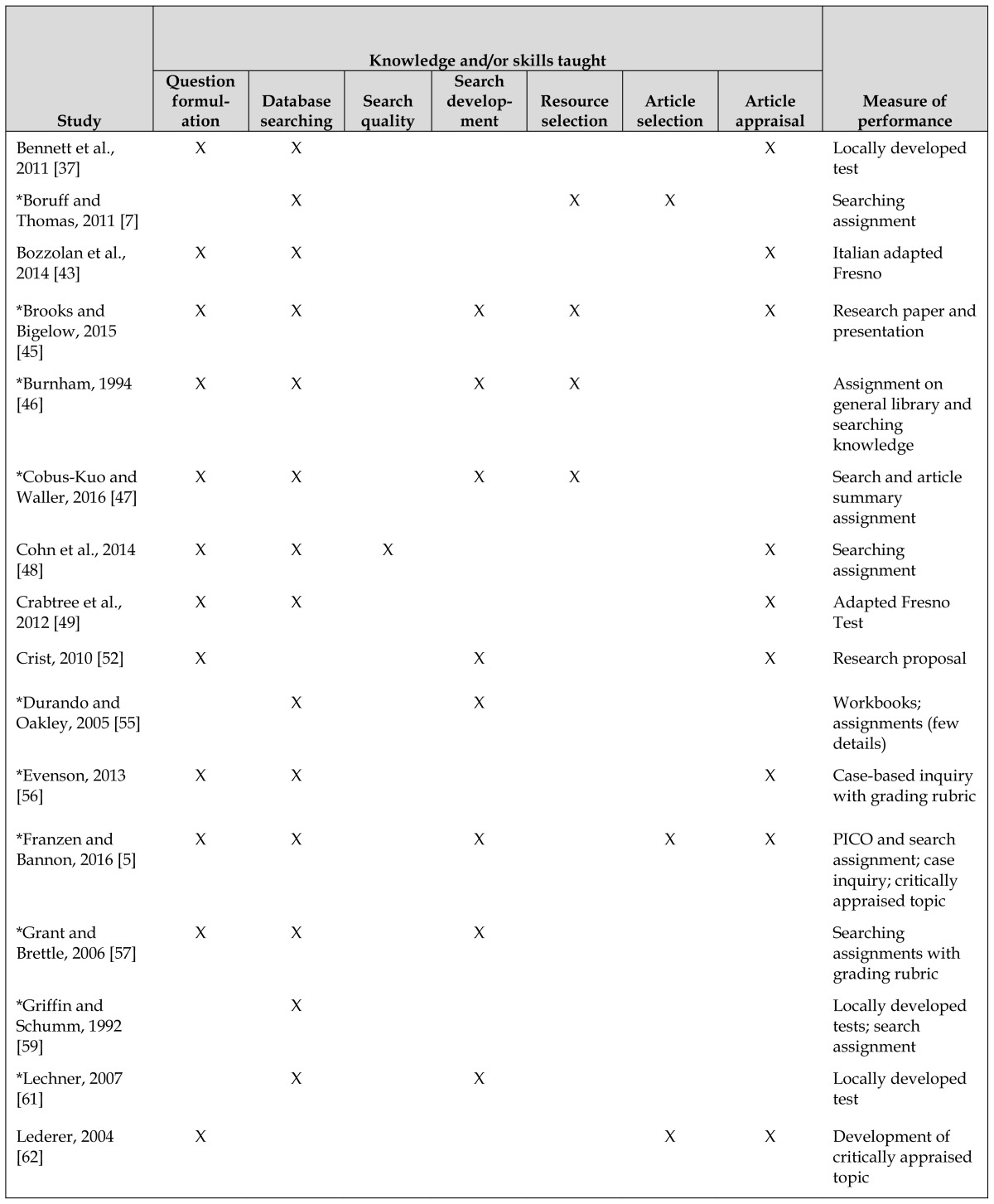
Librarian role in the study and assessment measures used

Study	Knowledge and/or skills taught							Measure of performance

	Question formulation	Database searching	Search quality	Search development	Resource selection	Article selection	Article appraisal	
Bennett et al., 2011 [37]	X	X					X	Locally developed test
*Boruff and Thomas, 2011 [7]		X			X	X		Searching assignment
Bozzolan et al., 2014 [43]	X	X					X	Italian adapted Fresno
*Brooks and Bigelow, 2015 [45]	X	X		X	X		X	Research paper and presentation
*Burnham, 1994 [46]	X	X		X	X			Assignment on general library and searching knowledge
*Cobus-Kuo and Waller, 2016 [47]	X	X		X	X			Search and article summary assignment
Cohn et al., 2014 [48]	X	X	X				X	Searching assignment
Crabtree et al., 2012 [49]	X	X					X	Adapted Fresno Test
Crist, 2010 [52]	X			X			X	Research proposal
*Durando and Oakley, 2005 [55]		X		X				Workbooks; assignments (few details)
*Evenson, 2013 [56]	X	X					X	Case-based inquiry with grading rubric
*Franzen and Bannon, 2016 [5]	X	X		X		X	X	PICO and search assignment; case inquiry; critically appraised topic
*Grant and Brettle, 2006 [57]	X	X		X				Searching assignments with grading rubric
*Griffin and Schumm, 1992 [59]		X						Locally developed tests; search assignment
*Lechner, 2007 [61]		X		X				Locally developed test
Lederer, 2004 [62]	X					X	X	Development of critically appraised topic
Lewis et al., 2016 [63]	X	X					X	K-REC
Portney, 2004 [70]	X						X	Development of critically appraised topic
*Raghavendra, 2009 [71]	X	X					X	Development of critically appraised topic
*Reynolds, 2010 [74]	X	X					X	Development of researchable clinical question; development of critically appraised topic
Sabus, 2008 [75]	X	X					X	Presentation of case-based inquiry
Scott et al., 2011 [76]	X	X					X	Poster presentation of case-based inquiry
*Thomas et al., 2012 [10]	X	X					X	Case-based inquiry with grading rubric
Van Moorsel 2005 [85]		X			X		X	Locally developed test; research presentations
*Villeneuve and Maranda, 2005 [86]	X	X			X			Case-based inquiry
Vogel, 2012 [87]	X	X	X					Searching assignment

* Librarian involvement.

## DISCUSSION

The forty included studies revealed a wide variety of attitude and performance measures being used in rehabilitation sciences student contexts. The published self-report surveys are worth investigating for adaptation in library contexts; however, a deeper investigation and discussion of these measures is beyond the intended scope of this review. These measures may provide guidance for librarians who want valid and reliable ways of understanding students’ perceptions of IL teaching and learning.

Of the validated tests of performance, the Adapted Fresno Test [51] and the Modified Fresno Test [90] have the most potential for instructional librarians to incorporate into their own assessments, as they have already been adapted to occupational therapy and physical therapy and have components for evaluating the first two steps of the EBP cycle.

This review did not find an example study using the Modified Fresno Test (other than the validation study), but it was included here as a suggested assessment measure for those teaching physical therapy students. Unfortunately, there is no validated English version for speech-language pathology. The benefit of the adapted or Modified Fresno Test is that it evaluates a real-life application of knowledge and skills [91], testing the student’s ability to take a case through the five EBP steps. One drawback of the adapted or Modified Fresno Test is that these measures were originally designed to be taken as a stand-alone exam, where the student is asked to describe how to search instead of providing an actual search history. As such, the adapted or Modified Fresno Test equates describing how to search to actually using a database and does not test the student’s ability to apply the knowledge and skills that librarians teach in IL instruction.

The K-REC test [65], while based on the Fresno Test, primarily tests knowledge and does not cover IL knowledge well. The CCTST [42] is a commercially available test and does not directly test EBP knowledge. The only IL standardized test included in our review, the RRSA [73], was not used in a course-integrated instruction setting, and it is unclear if it tests the effectiveness of IL instruction in EBP contexts.

The large number of assignments that focused on IL knowledge and skills provide many examples for librarians designing their own assignments for use in courses. Course assignments are an excellent method for librarians to become involved in the assessment cycle, especially when class time is limited. While time-consuming to develop, a valid and reliable grading rubric is one way for librarians to give more rigor to assignments used as assessment measures [92].

Our review did not find any examples of grading rubrics that measure skills in a meaningful way and are ready for use by interested librarians. Thomas et al. used a comprehensive and instructive EBP reference model [93] as a grading rubric for their EBP case-inquiry, but it would be impractical for librarians to use for grading assignments due to its complexity [10]. Grant and Brettle as well as Evenson used simple yes/no rubrics [56, 57]. The adaptation of the VALUE rubric to the graduate physical therapy student context by Turbow and Evener might be the most useful [82], as it assessed multiple skills in the EBP cycle and could be widely adopted by librarians who are grading case-based inquiries, critically appraised topics, or research project assignments. However, librarians using the VALUE rubric would most likely need to create more detailed requirements for the points awarded for each category in order to make it an effective assessment tool. It is our opinion that more studies are needed to verify the VALUE rubric’s reliability and validity in health sciences and graduate student contexts.

The fact that all but one of the included studies involving librarians used some measure of performance suggests that health sciences librarians are doing performance-based assessment. Most of these assessment measures were course assignments; therefore, their impact on learning can be more difficult than tests to quantify, unless they are graded in a systematic manner. The use of grading rubrics, as discussed above, and clearly defined learning outcomes aligned with these rubrics would allow better demonstration of the impact of assignments.

The fact that the majority of the included studies described course-integrated instruction suggests that a place has already been made in these curricula for EBP instruction and the accompanying IL knowledge and skills. We had hoped to make a connection between the learning outcomes of a course, the knowledge and skills taught by the instructional activities, and the assessment measures used in order to better understand how the knowledge and skills were assessed. However, most of the studies did not describe in sufficient detail all three components to make these connections. Instead, as it emerged that IL skills were often taught or stated as outcomes in courses where librarians were not mentioned, we collated what knowledge and skills were taught and by whom.

It was striking to discover the number of courses that reported learning outcomes that addressed searching and database use but that did not have a librarian involved in the course. This trend suggests that there are still barriers to instructors recognizing the value and expertise of librarians in EBP instruction. As experts in IL, librarians can model the proper knowledge and skills, particularly in the first three EBP steps, to help future clinicians reach eventual expertise and behavior in clinical practice [15].

With course learning outcomes already aligned with IL outcomes, there are many opportunities for librarians to work with instructors to develop course content and assessments to better teach to these outcomes, if instructors are open to this collaboration. In cases where the librarian is not involved at all, the time has already been made in the curriculum and course instructors may be open to assistance from the librarian expert in designing and teaching the content. In cases where the librarian is involved in the instruction, but not the assessment, there may be an assignment or other assessment measure that the librarian could use to assess IL knowledge and skills. With some modifications, portions of the adapted or Modified Fresno Test could be used by librarians to evaluate question formulation and database searching more effectively. Librarians could work with instructors to integrate the entire adapted or Modified Fresno Test into the course assessment. Another option would be for librarians to use the VALUE rubric as modified by Turbow and Evener [82] with existing assignments.

The limitations of our study were as follows: It was possible that we missed relevant studies due to the large and ever-changing body of literature. We also did not contact authors for additional data in cases where studies lacked detail about such points as the involvement of a librarian or specific learning objectives.

This review confirmed for us that useful assessment tools in EBP instruction take time to develop and implement, on the part of the librarian and in the context of larger course programs. Assessment measures that test knowledge can be useful, especially if that is the only measure that a librarian can integrate into a course. However, knowledge tests will not evaluate whether instructional content such as hands-on practice with question formulation and database searching is effective in skill development. Assessments that measure skills, such as grading rubrics or the adapted or Modified Fresno Test, better indicate the effectiveness of instruction on student learning. Future areas of research could focus on validating modifications of the adapted or Modified Fresno Test that better assess searching skills and on further validating the VALUE rubric in EBP contexts.

Health sciences librarians spend a great deal of time developing and delivering instruction, and it is important to find measures that assess this instruction in an authentic way. More studies need to be done on the long-term retention of IL knowledge and skills. Given the lifelong learning characteristics of rehabilitation sciences professional programs, measuring long-term knowledge retention and skill development is essential to proving the value of IL instruction.

## Supplemental File

Appendix MEDLINE search strategy
