# Supplementary material for: Assessment of knowledge and skills in information literacy instruction for rehabilitation sciences students: a scoping review
Source: J Med Libr Assoc. 2018 Jan 2;106(1):15–37. doi: 10.5195/jmla.2018.227 (PMC5764589; doi:10.5195/jmla.2018.227)
Supplement: Appendix [file jmla-106-15-S001.pdf]

JMLA Journal of the Medical Library Association

SC

Supplemental content to
J Med Libr Assoc. Jan;106(1):dx.doi.org/10.5195/jmla.2018.227
www.jmla.mlanet.org
© Boruff, Harrison 2018

Assessment of knowledge and skills in information literacy instruction for rehabilitation sciences students: a scoping review

Jill T. Boruff, AHIP; Pamela Harrison

APPENDIX

MEDLINE search strategy

1	Occupational Therapy/
2	occupational therapy.ti,ab.
3	Physical Therapists/ or Physical Therapy Specialty/
4	physical therapy.ti,ab.
5	physiotherapy.ti,ab.
6	Speech-Language Pathology/
7	(speech language pathology or speech therapy).ti,ab.
#8	Education, Nursing, Baccalaureate/ or Education, Nursing, Graduate/ or Education, Nursing/
9	Allied Health Occupations/
10	allied health.ti,ab.
#11	nursing.ti.
12	1 or 2 or 3 or 4 or 5 or 6 or 7 or 8 or 9 or 10 or 11
13	(librar* adj3 instruction).ti,ab.
14	Librarians/ or librarian*.ti,ab.
15	Computer User Training/ or Computer Literacy/ or Information Literacy/ or information literacy.ti,ab.
16	evidence based practice.ti,ab. or Evidence-Based Practice/ or Evidence-Based Nursing/
17	(fresno test or berlin questionnaire).ti,ab.
18	(database search* or pubmed search* or medline search* or literature search*).ti,ab.
19	question formulation.ti,ab.
20	((information or literature) adj retrieval).ti,ab.
21	((Searching or library or information) adj skills).ti,ab.
22	online searching.ti,ab.
23	bibliographic instruction.ti,ab.
24	information professional*.ti,ab.
25	or/13–24
26	(Curriculum or curricula or Education or academic or university or college or master* or undergraduate* or student* or graduate or doctoral).ti,ab.
27	12 and 25 and 26

Lines removed in search update.
